# Effects of Resveratrol on MCP-1/CCL2-Related Readouts in Preclinical Animal Models: A Systematic Review and Meta-Analysis

**DOI:** 10.3390/biomedicines14061285

**Published:** 2026-06-04

**Authors:** Yi-Lin Chiu, Shiue-Wei Lai, Sheng-Cheng Wu, Hsing-Fan Lai, Yi-Ying Wu, Tsung-Neng Tsai

**Affiliations:** 1 Graduate Institute of Biochemistry, College of Biomedical Science, National Defense Medical University, Taipei 114, Taiwan; yilin1107@mail.ndmutsgh.edu.tw; 2 Division of Hematology and Oncology, Department of Internal Medicine, Tri-Service General Hospital, National Defense Medical University, Taipei 114, Taiwan; xsurferlai@gmail.com (S.-W.L.); rq0922@gmail.com (Y.-Y.W.); 3 Department of Internal Medicine, School of Medicine, College of Medicine, National Defense Medical University, Taipei 114, Taiwan; 4 Division of Hematology and Oncology, Department of Internal Medicine, Tri-Service General Hospital Penghu Branch, Magong 880, Taiwan; gmsilent16@gmail.com; 5 Graduate Institute of Life Sciences, College of Biomedical Science, National Defense Medical University, Taipei 114, Taiwan; 809302001@mail.ndmutsgh.edu.tw; 6 Division of Cardiovascular Surgery, Department of Surgery, Tri-Service General Hospital, National Defense Medical University, Taipei 114, Taiwan

**Keywords:** resveratrol, MCP-1/CCL2, systematic review, meta-analysis, preclinical animal models

## Abstract

**Background**: Resveratrol is a plant-derived polyphenol with reported anti-inflammatory activity, and the MCP-1/CCL2 axis is a key mediator of monocyte recruitment and inflammatory tissue remodeling. Although individual preclinical studies have examined resveratrol effects on MCP-1/CCL2-related outcomes, the overall in vivo evidence has not been quantitatively synthesized. This systematic review and meta-analysis evaluated whether resveratrol treatment is associated with reduced MCP-1/CCL2-related inflammatory readouts in animal models. **Methods**: The protocol was registered in PROSPERO (CRD420261339126), and reporting followed the PRISMA 2020 statement. PubMed was searched from inception to 12 March 2026, with additional reference-list screening. Eligible studies were in vivo animal experiments comparing resveratrol-treated and control groups with extractable quantitative MCP-1/CCL2-related outcomes. Effect sizes were calculated as Hedges’ g with 95% confidence intervals and pooled using random-effects models fitted by restricted maximum likelihood. Subgroup, sensitivity, cumulative, influence, funnel-plot, dose meta-regression, and SYRCLE-based risk-of-bias analyses were conducted. **Results**: Twenty-seven studies contributing 29 analyzable datasets were included. The overall pooled effect was −3.74 (95% confidence interval, −4.50 to −2.98), indicating lower MCP-1/CCL2-related readouts in resveratrol-treated groups than in controls, with substantial heterogeneity (I2 = 78.9%). The negative association was driven mainly by rat and mouse datasets, whereas the piglet estimate was directionally opposite and the rabbit estimate came from a single dataset. Funnel-plot inspection suggested asymmetry, and dose meta-regression did not significantly explain between-study variation (slope = −0.17, *p* = 0.482). Leave-one-out and cumulative analyses indicated directional stability but did not resolve the underlying heterogeneity. **Conclusions**: These preclinical data indicate lower MCP-1/CCL2-related readouts after resveratrol treatment, but high heterogeneity, PubMed-only retrieval, and pharmacokinetic limitations limit direct clinical inference.

## 1. Introduction

Resveratrol has been studied as an anti-inflammatory polyphenol, but clinical effects remain inconsistent, partly because oral bioavailability is low and first-pass metabolism is rapid [1,2]. Mechanistically, resveratrol has been linked to activation of the AMPK/SIRT1 axis and downstream attenuation of NF-kappaB and NLRP3 inflammasome signaling pathways [3,4,5]. While experimental models frequently show reductions in pro-inflammatory cytokines such as TNF-alpha and IL-6, translation to human clinical trials has remained challenging. Clinical outcomes often reveal modest or variable anti-inflammatory effects, largely constrained by resveratrol’s low oral bioavailability, rapid first-pass metabolism, and marked inter-individual variability [6,7].

Despite these pharmacokinetic limitations, elucidating the precise molecular targets of resveratrol remains crucial, particularly in the context of immune cell recruitment during chronic inflammation. Monocyte chemoattractant protein-1 (MCP-1/CCL2) is a key chemokine involved in monocyte and macrophage recruitment to inflamed tissues. It signals through CCR2 and contributes to inflammatory amplification and tissue remodeling [8,9]. Secreted by diverse cell types including endothelial cells, fibroblasts, and podocytes, MCP-1 binds to its cognate receptor, CCR2, triggering downstream signaling cascades such as PI3K/Akt and MAPKs that amplify local inflammation and drive pathological tissue remodeling [10,11]. The MCP-1/CCL2-CCR2 axis is therefore recognized as an indispensable driver of macrophage-induced tissue destruction in a spectrum of conditions ranging from obesity-induced metabolic dysfunction to progressive renal injury [12,13].

Consequently, pharmacological blockade of the MCP-1/CCR2 axis has emerged as a promising, yet complex, therapeutic strategy, particularly in cardiovascular and renal diseases [14,15]. In preclinical models of atherosclerosis and diabetic nephropathy, inhibiting this axis reduces plaque volume, limits mesangial matrix expansion, and decreases macrophage burden [15,16]. However, in vivo interventions also reveal a double-edged-sword effect: although chronic inhibition can prevent adverse cardiac remodeling and heart failure, complete neutralization of MCP-1 during acute myocardial infarction may impair necessary necrotic clearance and wound healing [17,18,19]. Given the clinical complexities and systemic immunosuppression risks associated with direct monoclonal antibodies or CCR2 antagonists, there is growing interest in utilizing the broad gene-modulatory potential of resveratrol to therapeutically attenuate MCP-1. Yet, to date, a comprehensive quantitative synthesis evaluating the efficacy, dose dependency, and systemic consistency of resveratrol-mediated MCP-1 suppression across diverse in vivo models remains lacking.

Therefore, this systematic review and meta-analysis aimed to quantify the in vivo association between resveratrol administration and MCP-1/CCL2-related readouts across different physiological systems. By integrating current preclinical data and conducting sensitivity, subgroup, and meta-regression analyses, we sought to evaluate the consistency, dose relationship, and context dependency of this association, while clarifying the limits of translational inference from heterogeneous animal models.

## 2. Materials and Methods

### 2.1. Study Design and Reporting

**Figure 1:**
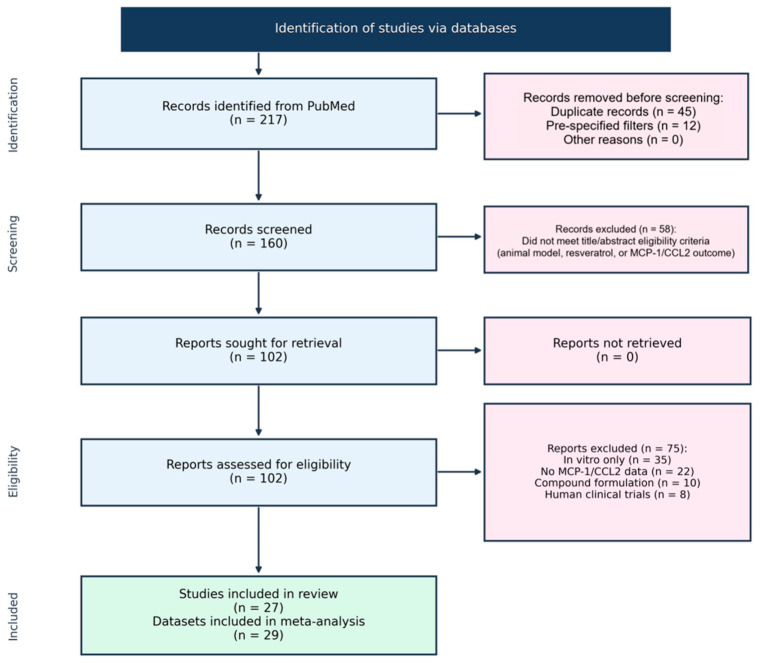
**Completed PRISMA 2020 flow diagram of study identification, screening, eligibility assessment, and inclusion.** In the flow diagram, blue boxes denote the identification, screening, and eligibility-assessment steps; pink boxes denote records or reports removed or excluded at each stage; the green box denotes the final studies and analyzable datasets included in the review and meta-analysis; arrows indicate the direction of record flow through the screen-ing process. Records were identified through PubMed database searching, followed by duplicate removal and pre-specified record-level filtering before manual title/abstract screening. Title/abstract exclusions reflected failure to meet the core animal model, resveratrol exposure, or MCP-1/CCL2-related outcome criteria. Full-text reports were excluded on the basis of in vitro-only design, absence of MCP-1/CCL2 outcome data, non-standard compound formulation, or human clinical-trial setting. No reports were unavailable for retrieval. The final dataset comprised 27 studies contributing 29 analyzable datasets to the quantitative synthesis.

This systematic review and meta-analysis was conducted and reported in accordance with the Preferred Reporting Items for Systematic Reviews and Meta-Analyses (PRISMA) 2020 statement. The review protocol was registered in PROSPERO under record ID CRD420261339126 (Effects of Resveratrol on MCP-1/CCL2-Related Inflammatory Responses in Preclinical Animal Models: A Systematic Review and Meta-analysis), with the record created on 12 March 2026 and last edited on 12 March 2026. A completed PRISMA 2020 flow diagram is provided as Figure 1, and the completed PRISMA 2020 checklist is provided as a supplementary file. Screening decisions, title/abstract exclusion categories, and full-text exclusion annotations were documented in Supplementary Worksheet S1.

### 2.2. Literature Search and Study Selection

The PROSPERO-registered search strategy specified searches in PubMed from database inception to 12 March 2026, with additional records identified through manual screening of the reference lists of included studies and relevant review articles. The representative full electronic strategy was built around three core concepts: resveratrol, MCP-1/CCL2-related terms, and animal/preclinical study terms. The representative PubMed strategy was defined as follows: ((“resveratrol” OR “trans-resveratrol” OR “3,5,4′-trihydroxystilbene”) AND (“MCP-1” OR “monocyte chemoattractant protein-1” OR “monocyte chemoattractant protein 1” OR “CCL2” OR “chemokine ligand 2” OR “C-C motif chemokine ligand 2”) AND (“animal” OR “animals” OR “mouse” OR “mice” OR “murine” OR “rat” OR “rats” OR “rodent” OR “rabbit” OR “rabbits” OR “preclinical” OR “model” OR “models”)). Records were exported in PubMed/NBIB format where applicable and screened in a stepwise manner through title/abstract review followed by full-text eligibility assessment. The automation category in the flow diagram refers to pre-specified record-level filters applied before manual screening; no study was excluded solely by an opaque machine-learning classifier. Studies were excluded if they were in vitro-only reports, lacked MCP-1/CCL2 outcome data, used non-comparable compound formulations, represented human clinical trials, or otherwise failed to provide an extractable quantitative comparison between resveratrol-treated and control groups. Screening decisions and exclusion annotations are summarized in Supplementary Worksheet S1. Because Embase, Web of Science, Scopus, and Cochrane Library were not searched, incomplete retrieval and database-selection bias cannot be excluded.

### 2.3. Keyword Extraction and Bibliometric Visualization

To provide a descriptive overview of the included corpus, the included PubMed records were imported into R (v 4.5.1) and converted to a bibliometrix-compatible data frame. Author- and index-derived keyword fields were parsed using a semicolon delimiter and standardized by harmonizing capitalization, abbreviations, and obvious synonyms. Keywords with a minimum frequency of at least 4 were retained to reduce sparsity. A co-occurrence matrix was constructed based on within-record co-appearance, visualized using a force-directed layout, and communities were detected with the Louvain algorithm. Keyword frequencies were summarized as a ranked bar plot of the top 15 terms and as a word cloud to represent relative term prominence (Supplementary Figure S1).

### 2.4. Data Extraction and Outcome Definition

For each eligible dataset, we extracted the study identifier, species, disease model, tissue or sample compartment, resveratrol dose, sample size, assay modality, and MCP-1/CCL2-related quantitative outcome values required to compute effect sizes. Continuous outcomes were captured as group mean, standard deviation, and sample size for resveratrol-treated versus control arms. Because included studies reported different outcome types, the synthesized endpoint was defined as an MCP-1/CCL2-related readout rather than a single expression measure. When multiple tissues, assay modalities, or experimental strata were reported within a single study, each was treated as a separate analyzable dataset. This approach preserved biologically relevant information but may introduce within-study dependency because datasets from the same experiment are likely to be correlated. Effect directions were encoded so that negative standardized mean differences indicated lower MCP-1/CCL2-related signal in the resveratrol group. The final analyzable study list is summarized in Supplementary Worksheet S2, and variable-level outcome typing is summarized in Supplementary Worksheet S3 [20,21,22,23,24,25,26,27,28,29,30,31,32,33,34,35,36,37,38,39,40,41,42,43,44,45,46].

### 2.5. Effect-Size Calculation and Primary Meta-Analysis

**Figure 2:**
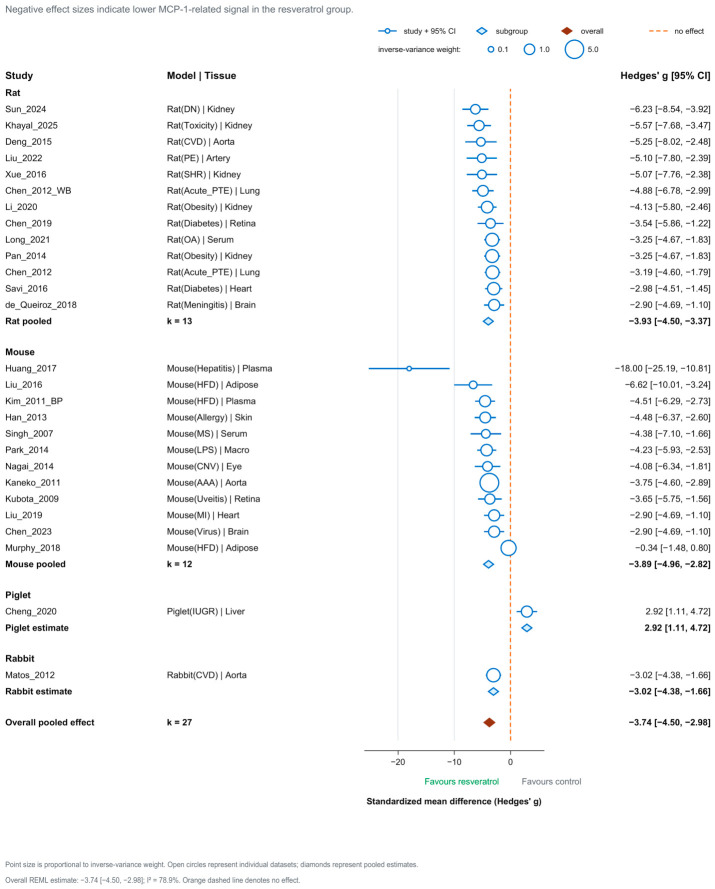
Forest plot of Hedges’ g for MCP-1-related outcomes in resveratrol-treated versus control groups. Negative values indicate lower MCP-1-related signal in the resveratrol group. Author-year study labels correspond to the included animal datasets [20,21,22,23,24,25,26,27,28,29,30,31,32,33,34,35,36,37,38,39,40,41,42,43,44,45,46]. Open blue circles and horizontal blue lines indicate individual dataset es-timates and 95% confidence intervals, with circle size proportional to inverse-variance weight; representative leg-end values are 0.1, 1.0, and 5.0. Blue diamonds indicate species-specific pooled or single-dataset estimates, the dark red diamond indicates the overall pooled estimate, and the orange dashed vertical line indicates the no-effect ref-erence. Rat and mouse categories are pooled subgroup estimates, whereas piglet and rabbit are shown as sin-gle-dataset species estimates. The overall REML estimate was −3.74 (95% CI, −4.50 to −2.98; I2 = 78.9%).

The primary effect size was the standardized mean difference, calculated as Hedges’ g, with 95% confidence intervals (CI). Study-specific effect sizes and sampling variances were synthesized using a random-effects meta-analysis fitted by restricted maximum likelihood (REML). Point estimates were weighted by inverse variance. Species-specific analyses were performed for the major taxonomic subgroups represented in the dataset. Rat and mouse categories contained multiple datasets and were summarized as pooled subgroup estimates, whereas piglet and rabbit categories each contained one dataset and were reported as single-dataset species estimates (Figure 2).

### 2.6. Dose Meta-Regression and Tissue-Level Subgroup Analysis

**Figure 3:**
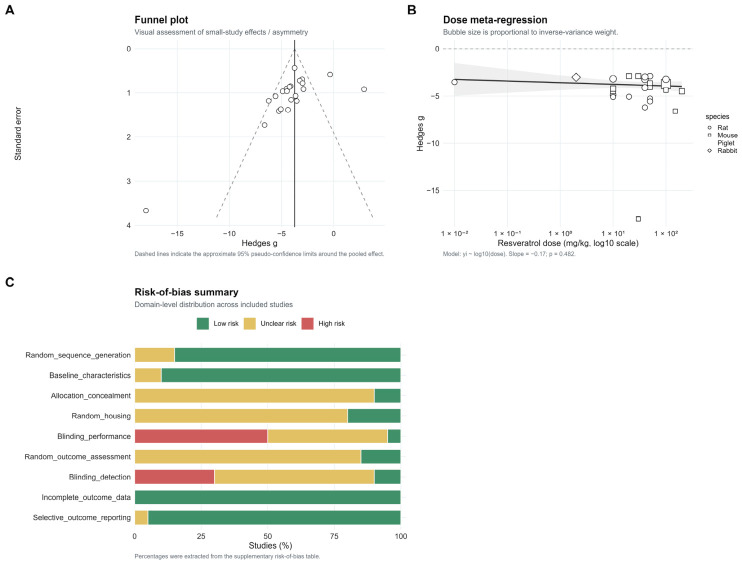
**Funnel plot, dose meta-regression, and domain-level risk-of-bias summary.** (**A**) Funnel plot of effect sizes against standard error. (**B**) Bubble plot of dose meta-regression examining the relationship between resveratrol dose and Hedges’ g on a log10 dose scale; bubble size reflects inverse-variance weight. (**C**) Risk-of-bias summary across included studies, presented as study percentages for each domain. Funnel limits indicate approximate 95% pseudo-confidence boundaries around the pooled estimate. Weighted meta-regression was performed on log10-transformed dose values; the reported slope was −0.17 with *p* = 0.482. Risk-of-bias values are shown as study percentages by domain.

**Figure 4:**
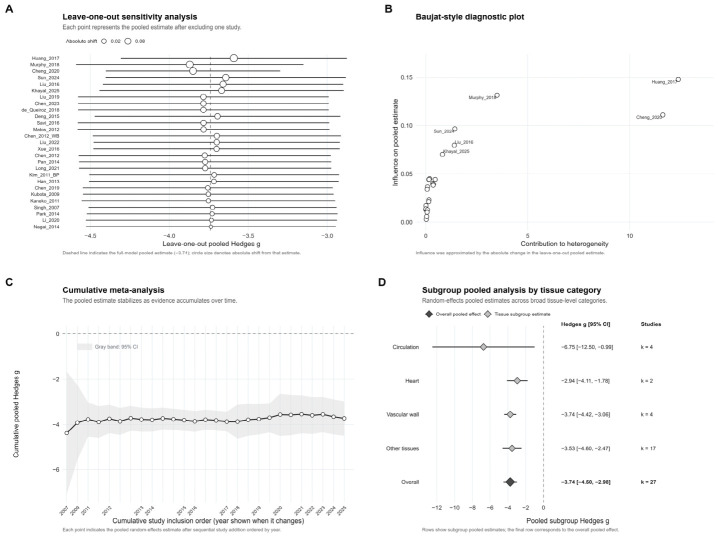
**Leave-one-out sensitivity, Baujat-style influence, cumulative meta-analysis, and tissue-category subgroup analyses.** (**A**) Leave-one-out sensitivity analysis showing the pooled estimate recalculated after sequential omission of each included dataset [20,21,22,23,24,25,26,27,28,29,30,31,32,33,34,35,36,37,38,39,40,41,42,43,44,45,46]. (**B**) Baujat-style diagnostic plot showing each study’s contribution to between-study heterogeneity and its influence on the pooled estimate. (**C**) Cumulative meta-analysis ordered by publication year. (**D**) Subgroup pooled analysis by tissue category, including circulation, heart, vascular wall, and other tissues. All pooled estimates are reported as Hedges’ g with 95% confidence intervals from random-effects models. Leave-one-out and cumulative analyses were based on repeated model refitting, and subgroup estimates were calculated within predefined tissue categories.

To assess whether treatment dose accounted for between-study variability, a meta-regression model was fitted using resveratrol dose as a moderator. Based on the analytical workflow shown in the figures, dose values were modeled on a log10 scale (Figure 3B). Extracted dose values used for the model are listed in Supplementary Worksheet S5. Additional subgroup meta-analyses were performed after grouping datasets into broad tissue categories, including circulation, heart, vascular wall, and other tissues (Figure 4D). Each subgroup was analyzed within the same random-effects framework used in the primary model.

### 2.7. Sensitivity, Cumulative, and Influence Analyses

Directional stability of the pooled estimate was examined using a leave-one-out approach in which the random-effects model was repeatedly refitted after sequential omission of each dataset (Figure 4A). Cumulative meta-analysis was conducted by ordering studies chronologically and recalculating the pooled estimate after iterative addition of each study (Figure 4C). Influence diagnostics were visualized using a Baujat-style plot to display each dataset’s contribution to heterogeneity and its influence on the pooled estimate simultaneously (Figure 4B).

### 2.8. Funnel-Plot Inspection and Risk-of-Bias Assessment

Potential small-study effects or asymmetry were assessed visually using a funnel plot of effect size versus standard error, with pseudo-confidence limits drawn around the pooled estimate (Figure 3A). Because the available figure set did not include the full effect-size table required to implement a reproducible formal asymmetry test, inference regarding publication bias was limited to qualitative visual inspection. This limitation is important because funnel-plot asymmetry may reflect publication bias, true biological heterogeneity, small-study effects, assay differences, or dependency among effect sizes rather than one single mechanism. Worksheet-level risk-of-bias summaries are provided in Supplementary Worksheet S4. Risk of bias was evaluated across nine methodological domains: random sequence generation, baseline characteristics, allocation concealment, random housing, blinding of performance, random outcome assessment, blinding of detection, incomplete outcome data, and selective outcome reporting. Domain-level judgments were summarized as percentages classified as low, unclear, or high risk (Figure 3C).

## 3. Results

### 3.1. Study Selection

A structured literature-screening workflow identified 217 records from database searching, of which 45 duplicates and 12 records flagged by pre-specified record-level filters were removed before manual screening. After title/abstract review of 160 records, 58 records were excluded because they did not meet one or more core eligibility criteria, including an in vivo animal model, resveratrol exposure, or an MCP-1/CCL2-related outcome. One hundred and two full-text reports were then assessed for eligibility. Seventy-five full-text articles were excluded because they were in vitro-only studies, lacked MCP-1/CCL2 outcome data, used non-standard compound formulations, or represented human clinical trials. Ultimately, 27 studies contributing 29 analyzable datasets were included in the meta-analysis (Figure 1; Supplementary Worksheets S1 and S2). Descriptive keyword analysis indicated that the resveratrol-MCP-1 literature spans inflammatory and organ-specific disease contexts rather than a single experimental niche (Supplementary Figure S1).

### 3.2. Random-Effects Meta-Analysis Showed a Broad Preclinical Association Between Resveratrol Exposure and Lower MCP-1-Related Signal

The primary random-effects meta-analysis demonstrated a significant overall negative pooled effect, indicating lower MCP-1-related readouts in resveratrol-treated groups relative to controls (Figure 2). The overall pooled standardized mean difference was −3.74 (95% CI, −4.50 to −2.98), consistent with a broad negative association between resveratrol exposure and MCP-1-associated inflammatory output across the included datasets. However, heterogeneity was substantial (I2 = 78.9%), indicating marked between-study variability in effect magnitude.

Species-stratified analysis showed that the dominant evidence arose from rat and mouse models, both of which exhibited strongly negative pooled estimates (Figure 2). The rat subgroup yielded a pooled effect of −3.93 (95% CI, −4.50 to −3.37), whereas the mouse subgroup showed a pooled effect of −3.89 (95% CI, −4.96 to −2.82). In contrast, the single piglet dataset showed an effect in the opposite direction, with a positive Hedges’ g of 2.92 (95% CI, 1.11 to 4.72), while the single rabbit dataset remained negative at −3.02 (95% CI, −4.38 to −1.66). Because piglet and rabbit categories each contained only one dataset, these estimates should not be interpreted as subgroup pooled effects. These findings indicate that the aggregate inverse association between resveratrol and MCP-1 was driven primarily by rodent datasets and was not uniformly represented across all species categories.

Notably, individual-study estimates varied widely, ranging from near-null effects to very large negative effects (Figure 2). This dispersion supports the interpretation that resveratrol-associated reductions in MCP-1-related readouts were directionally recurrent but context dependent in magnitude. Accordingly, the most defensible conclusion from the pooled analysis is that resveratrol was broadly associated with reduced MCP-1-related inflammatory signaling across preclinical models, rather than exerting a fixed effect size irrespective of model system.

### 3.3. Dose Did Not Explain Between-Study Variation, and Asymmetry Was Partly Driven by Influential Studies

Visual inspection of the funnel plot suggested asymmetry, with a small number of datasets positioned far from the main concentration of effect sizes (Figure 3A). Because a reproducible formal asymmetry test could not be performed from the available figure set alone, these data support a cautious interpretation of potential small-study effects or influence from extreme observations rather than a definitive conclusion regarding publication bias alone. Meta-regression further indicated that heterogeneity was not explained by resveratrol dose within the sampled preclinical range (Figure 3B). When dose was modeled on a log10 scale, the fitted slope was shallow and non-significant (slope = −0.17, *p* = 0.482), suggesting that the observed suppression of MCP-1 was not linearly dose dependent in the pooled dataset. The dose inputs underlying this analysis are listed in Supplementary Worksheet S5. This result argues against dose as the principal source of between-study variability and points instead to alternative contributors such as species, disease context, tissue compartment, assay modality, formulation, route of administration, or study quality. The risk-of-bias summary showed a mixed methodological profile across studies (Figure 3C). Domain-level worksheet summaries are provided in Supplementary Worksheet S4. Several domains, including incomplete outcome data and selective outcome reporting, were predominantly rated as low-risk. By contrast, allocation concealment and random housing were frequently categorized as unclear, and performance/detection blinding showed a larger proportion of unclear or high-risk judgments. These patterns indicate that although many studies reported core experimental outcomes adequately, procedural safeguards against bias were incompletely documented in several domains.

### 3.4. Sensitivity, Influence, and Cumulative Analyses Indicated Directional Stability of the Pooled Association

Leave-one-out analysis showed that removal of any single study did not reverse the direction of the overall pooled estimate (Figure 4A). Across all refitted models, the pooled Hedges’ g remained consistently negative, indicating that the main association was not solely driven by one dataset. This directional stability is important given the wide dispersion of study-level effects observed in the primary forest plot, but it does not eliminate the substantial biological and methodological heterogeneity.

The Baujat-style diagnostic plot nonetheless identified several studies as outsized contributors to heterogeneity and pooled-effect displacement (Figure 4B), particularly the datasets reported by [31,34,36,40,45,46]. These studies likely account for a substantial fraction of the observed asymmetry and between-study variance, consistent with the high I2 in the primary meta-analysis. Their identification does not invalidate the overall association, but it indicates that the pooled estimate is shaped disproportionately by a subset of datasets with extreme magnitude, unusual variance structure, or both.

Cumulative meta-analysis showed that the pooled effect stabilized in the negative range as evidence accumulated over time (Figure 4C). After the early studies were incorporated, subsequent study addition produced only modest fluctuations around an already negative pooled estimate, supporting temporal stability in the direction of effect. This pattern suggests that the central conclusion—lower MCP-1-related signal in resveratrol-treated preclinical systems—was not a late-emerging artifact driven only by the most recent reports.

### 3.5. Tissue-Level Subgroup Analysis Suggested Broad but Non-Uniform Suppression Across Biological Compartments

Subgroup analysis by tissue category demonstrated that the negative resveratrol-associated effect extended across multiple biological compartments (Figure 4D). The vascular wall subgroup showed a pooled effect of −3.74 (95% CI, −4.42 to −3.06), closely matching the overall estimate, whereas the heart subgroup showed a somewhat less extreme but still clearly negative effect of −2.94 (95% CI, −4.11 to −1.78). The “other tissues” category also remained significantly negative at −3.53 (95% CI, −4.60 to −2.47).

The circulation subgroup exhibited the most extreme pooled negative estimate, −6.75 (95% CI, −12.50 to −0.99), but also the broadest confidence interval (Figure 4D). This wider interval implies lower precision and possibly greater contextual heterogeneity in circulating MCP-1 measurements than in tissue-localized readouts. Nonetheless, because all major tissue categories retained negative pooled estimates, the data support the interpretation that resveratrol-associated attenuation of MCP-1 is not restricted to a single organ system. Instead, the effect appears to extend across renal, vascular, cardiac, and circulating inflammatory contexts, albeit with variable magnitude and certainty.

## 4. Discussion

This systematic review and meta-analysis shows that resveratrol exposure is broadly associated with lower MCP-1/CCL2-related inflammatory readouts in vivo. Across diverse preclinical contexts, particularly rat and mouse disease models, resveratrol administration was generally associated with reduced MCP-1-related signals. However, the magnitude of this association varied substantially across species, tissue compartments, assay modalities, formulations, and influential datasets, and it was not significantly explained by treatment dose. Therefore, the most defensible interpretation is a directionally consistent but context-dependent preclinical association, rather than definitive evidence of a uniform pharmacologic effect.

The biological plausibility of this association is supported by prior work linking MCP-1 to monocyte chemotaxis and macrophage infiltration in inflamed tissues, a process that can amplify chronic inflammation and plaque formation [14,47]. Resveratrol has also been reported to modulate AMPK/SIRT1, NF-kappaB, oxidative-stress, and inflammasome-related pathways [3,4,5,48]. However, these pathways were not systematically extracted or meta-analyzed in the present review. Accordingly, the mechanistic interpretation should be regarded as biologically plausible and literature-consistent, but not directly demonstrated by the pooled MCP-1/CCL2 dataset itself.

The translational relevance of MCP-1/CCL2 modulation remains important but should be framed cautiously. In cardiovascular and renal systems, high MCP-1 levels have been associated with endothelial dysfunction, mesangial matrix expansion, fibrosis, cardiovascular mortality, and renal decline in diabetic nephropathy [13,49,50]. The present findings therefore support continued investigation of the CCL2-CCR2 axis as an inflammatory target, consistent with early-stage translational interventions involving CCR2 antagonists or CCL2 inhibitors [51,52]. Nevertheless, the evidence synthesized here does not establish reproducible therapeutic efficacy in humans. At most, it provides a preclinical rationale for testing whether resveratrol, optimized formulations, or better-characterized analogs can modulate MCP-1/CCL2-related inflammatory biology under defined experimental conditions, particularly given the known limitations in oral bioavailability, inter-individual metabolic variability, and formulation-dependent delivery [53,54,55,56].

The primary strength of this study lies in the integration of diverse in vivo datasets and the use of leave-one-out, cumulative, and influence diagnostics to evaluate directional stability. However, several limitations must be acknowledged. First, the meta-analysis revealed substantial statistical heterogeneity (I2 = 78.9%), reflecting variation in animal species, disease models, tissue compartments, assay modalities, routes of administration, formulations, and study quality. This heterogeneity limits the precision of the pooled effect and precludes a universal therapeutic threshold. Second, the search was restricted to PubMed plus reference-list screening; therefore, studies indexed only in Embase, Web of Science, Scopus, Cochrane Library, or other databases may have been missed. Third, the pooled standardized mean difference was very large, which may partly reflect small sample sizes, assay-normalization differences, selective reporting, or influential datasets rather than a single biological effect. Fourth, the analysis treated multiple tissues, assays, or strata from the same study as separate datasets, so within-study dependency cannot be fully excluded.

From a methodological perspective, this review should be interpreted in light of current reporting and synthesis guidance. PRISMA 2020 and PRISMA-S emphasize transparent reporting of information sources, search strategies, eligibility decisions, and reproducible search methods, supporting the explicit PubMed-only limitation and flow-diagram clarification added here [57,58]. In addition, funnel-plot asymmetry cannot be equated with publication bias, and conventional asymmetry tests for standardized mean differences may be unstable when heterogeneity and effect-size dependency are present [59,60]. Preclinical animal meta-analyses also require attention to model-level heterogeneity and dependent outcomes; multilevel or robust-variance approaches may be preferable when sufficient study clusters and covariance information are available [61,62]. Because no formal adapted GRADE-like certainty assessment was performed, the present findings should be considered hypothesis-generating preclinical evidence with limited translational certainty rather than definitive therapeutic proof [63].

## 5. Conclusions

In conclusion, resveratrol exposure was associated with lower MCP-1/CCL2-related inflammatory readouts across preclinical animal models, particularly in rodent datasets. However, the evidence remains heterogeneous, preclinical, and methodologically variable, with no significant dose–response relationship and single-dataset estimates for non-rodent species. These findings support further mechanistic and translational studies, but they do not establish resveratrol as a reliable therapeutic inhibitor of the MCP-1/CCL2-CCR2 axis in humans. Future work should prioritize multi-database evidence retrieval, standardized MCP-1/CCL2 outcome reporting, dependency-aware meta-analytic models, and strategies that address the poor oral bioavailability and pharmacokinetic variability of resveratrol before clinical application is inferred.

## Data Availability

The data supporting this study are available within the article and its Supplementary Materials. Additional information is available from the corresponding author upon reasonable request.

## Supplementary Materials

The following supporting information can be downloaded at https://www.mdpi.com/article/10.3390/biomedicines14061285/s1, Supplementary Worksheet S1: Screening decisions, title/abstract exclusion categories, and full-text exclusion annotations. Supplementary Worksheet S2: The 29 analyzable datasets included in the meta-analysis. Supplementary Worksheet S3: Variable-level outcome types used for effect-size extraction. Supplementary Worksheet S4: Domain-level risk-of-bias summary. Supplementary Worksheet S5: Extracted resveratrol dose values used in the dose me-ta-regression analysis. Supplementary Figure S1: Keyword co-occurrence and frequency summaries of the included literature [20,21,22,23,24,25,26,27,28,29,30,31,32,33,34,35,36,37,38,39,40,41,42,43,44,45,46]. The completed PRISMA 2020 checklist is provided as an additional supplementary file.

## Abbreviations

Akt, protein kinase B; AMPK, AMP-activated protein kinase; CCL2, C-C motif chemokine ligand 2; CCR2, C-C chemokine receptor 2; CI, confidence interval; IL-6, interleukin-6; I2, I-squared heterogeneity statistic; MAPKs, mitogen-activated protein kinases; MCP-1, monocyte chemoattractant protein-1; NBIB, PubMed bibliographic citation file; NF-kappaB, nuclear factor kappa B; NLRP3, NOD-like receptor family pyrin domain-containing 3; NSTC, National Science and Technology Council; PI3K, phosphoinositide 3-kinase; PRISMA, Preferred Reporting Items for Systematic Reviews and Meta-Analyses; PROSPERO, International Prospective Register of Systematic Reviews; REML, restricted maximum likelihood; SIRT1, sirtuin 1; TNF-alpha, tumor necrosis factor-alpha.
