# Supplementary material for: Effects of Resveratrol on MCP-1/CCL2-Related Readouts in Preclinical Animal Models: A Systematic Review and Meta-Analysis
Source: Biomedicines. 2026 Jun 4;14(6):1285. doi: 10.3390/biomedicines14061285 (PMC13297441; doi:10.3390/biomedicines14061285)
Supplement: Supplementary file 1 [file biomedicines-14-01285-s001.zip › SF1.pdf]

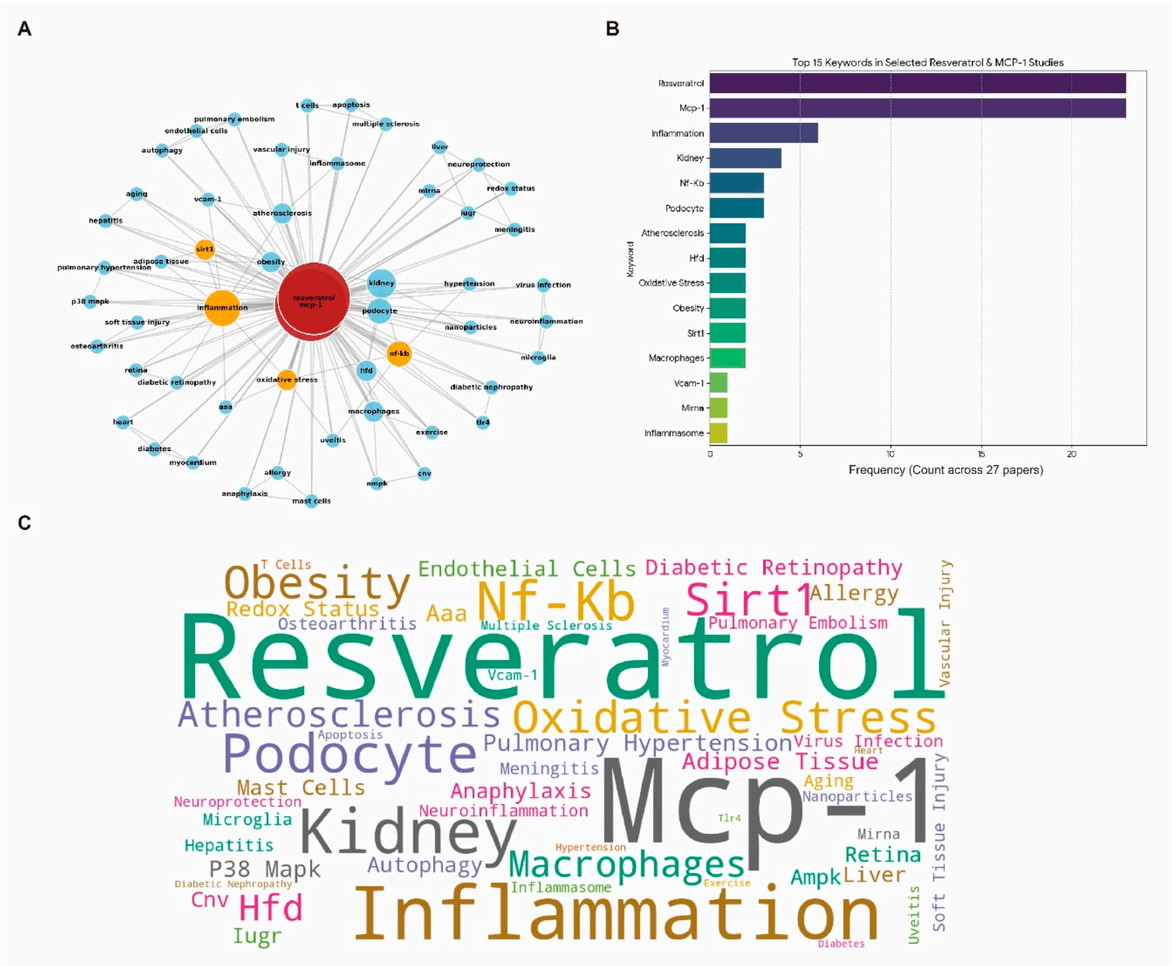

Supplementary Figure S1. Keyword co-occurrence and frequency summaries of the included literature. [20–46] (A) Network visualization of co-occurring keywords extracted from the included studies. "Resveratrol/MCP-1" is positioned at the center of the network and is linked to inflammation-related, renal, vascular, oxidative-stress, and signaling terms, including kidney, podocyte, NF-kappaB, oxidative stress, obesity, and SIRT1. (B) Bar plot showing the frequency of the top 15 keywords across the selected studies. (C) Word-cloud representation of recurring terms within the included corpus, with larger words indicating greater frequency.
